# Supplementary material for: Histone Variant H2A.J Is Enriched in Luminal Epithelial Gland Cells
Source: Genes (Basel). 2021 Oct 22;12(11):1665. doi: 10.3390/genes12111665 (PMC8619693; doi:10.3390/genes12111665)
Supplement: Supplementary file 1 [file genes-12-01665-s001.zip › SupplementaryFigures_H2A.JLuminalEpithelial_RedonEtAl.pdf]

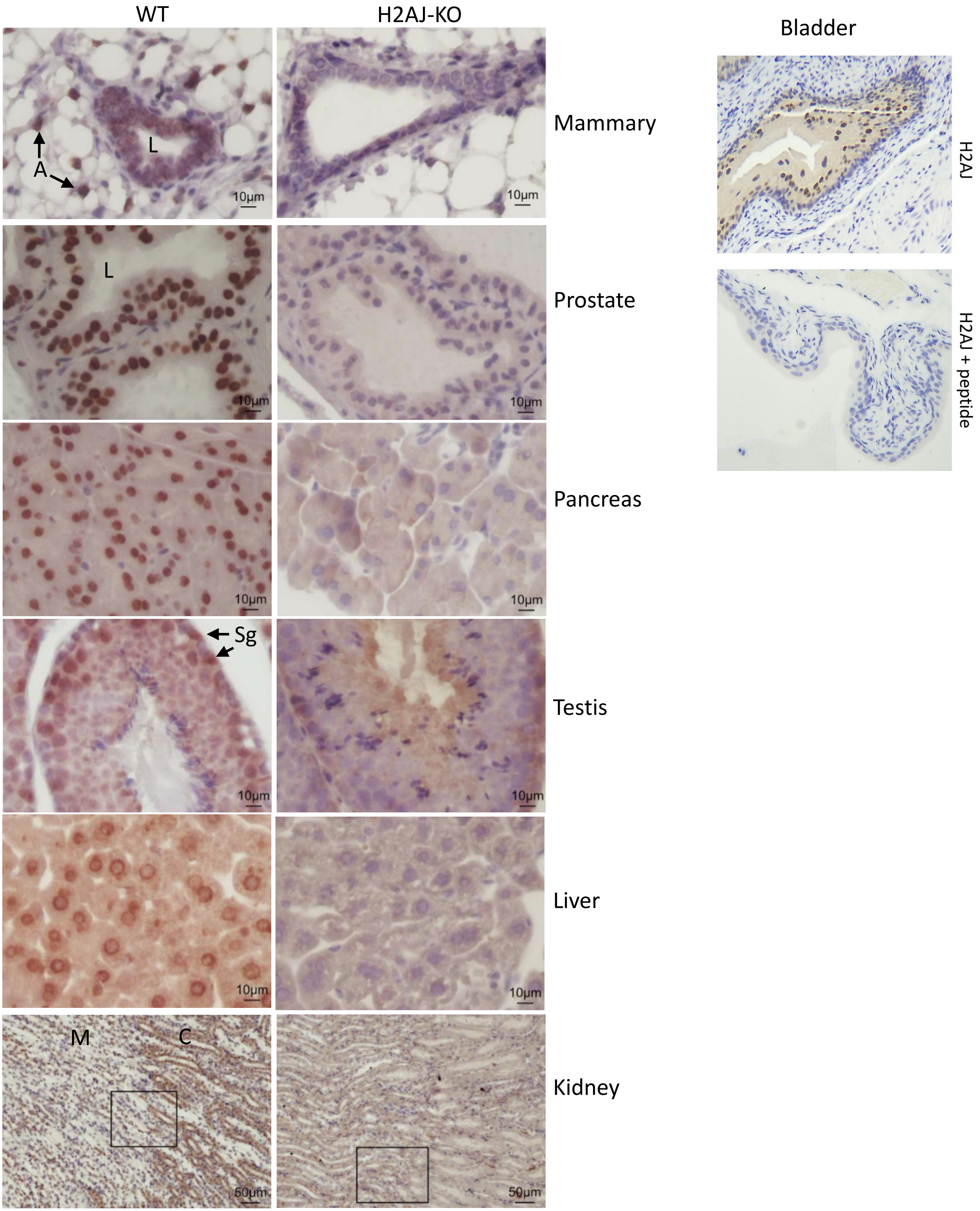

Supplementary Figure S1. Specificity of anti-H2A.J staining demonstrated by parallel immunohistochemical detection of H2A.J in C57BL6-N WT and H2A.J-KO tissue sections for the indicated mouse organs. L=lumen; A=adipocytes, Sg=spermatogonia, M=medulla, C=cortex. The right panels show an example of specificity by competition of the H2A.J signal by an excess of the C-terminal H2A.J-specific peptide that was used as the immunogen to prepare the antibody.

A

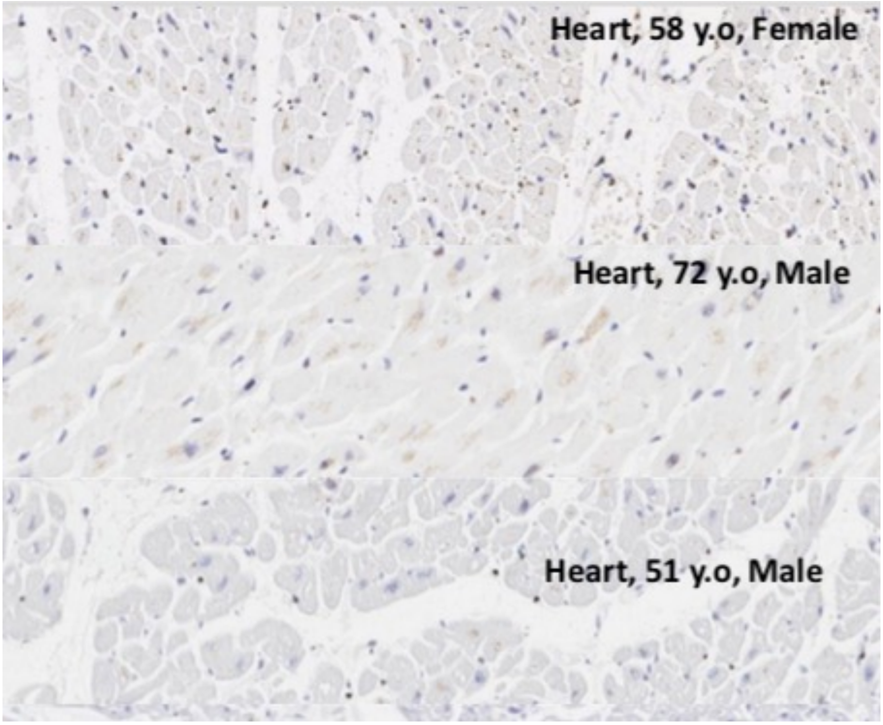

B

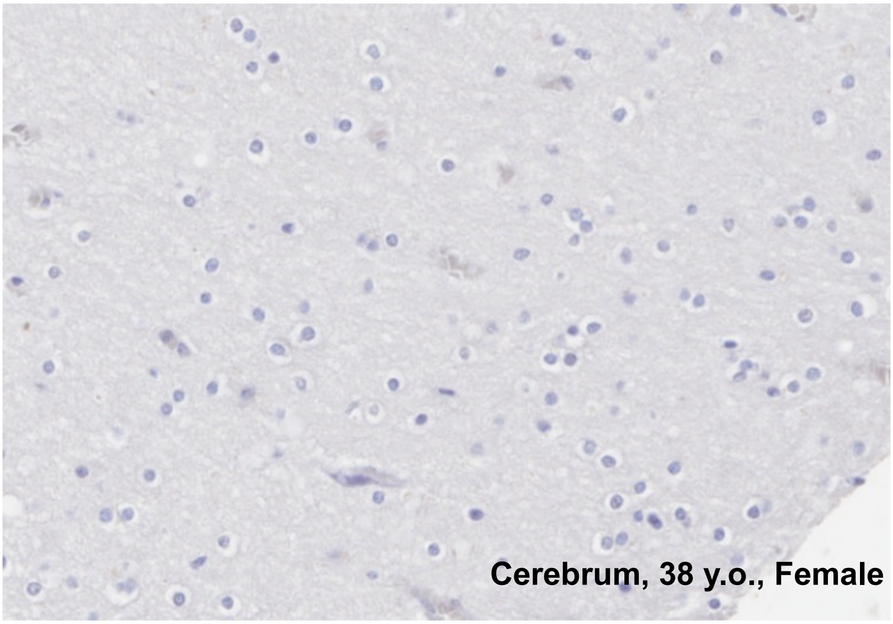

Supplementary Figure S2. Human heart and cerebrum tissue sections show low levels of H2A.J by immunohistochemical staining.

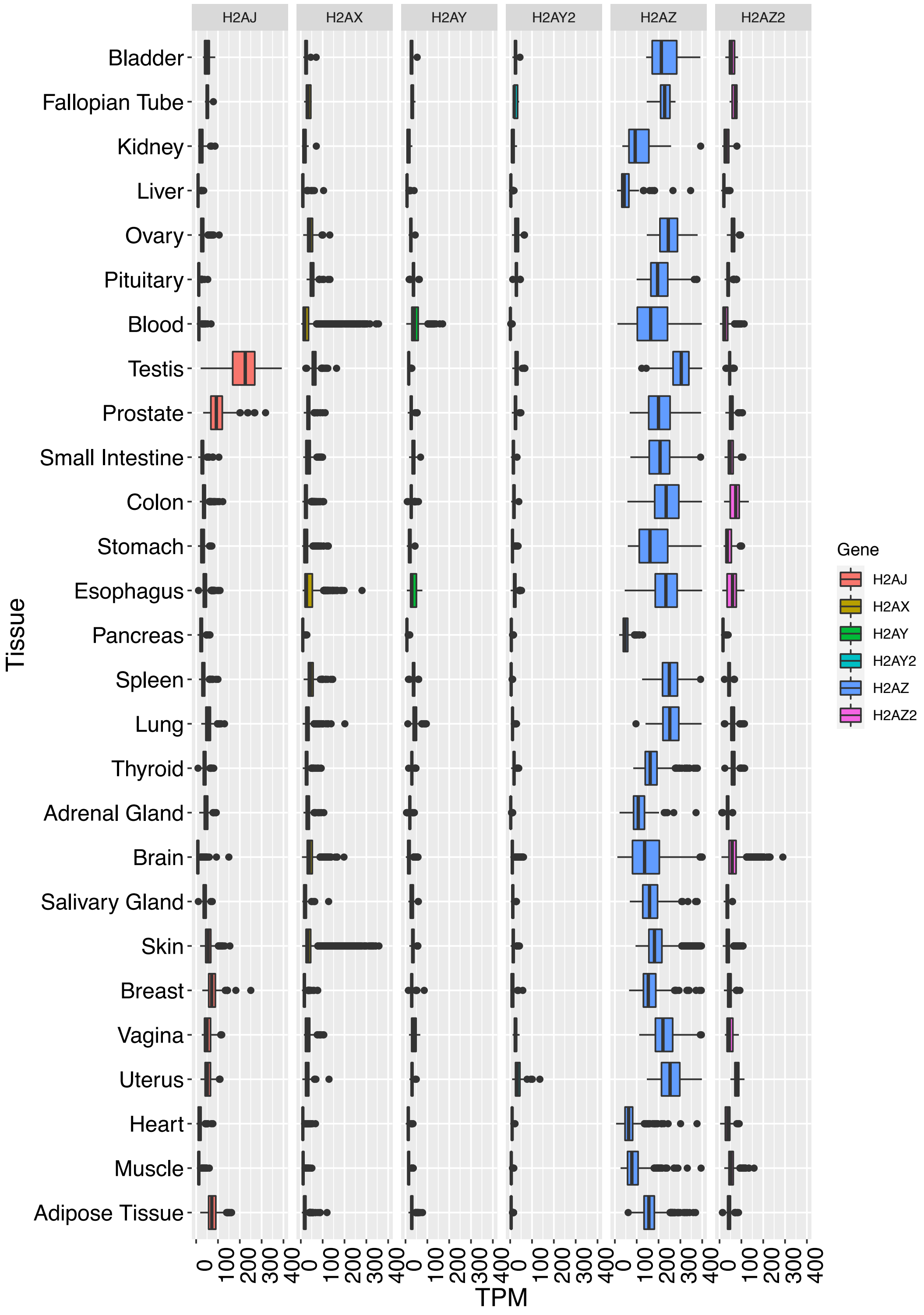

Supplementary Figure S3. RNA levels (TPM) for the indicated histone variant genes in 27 human tissues from GTEx data.

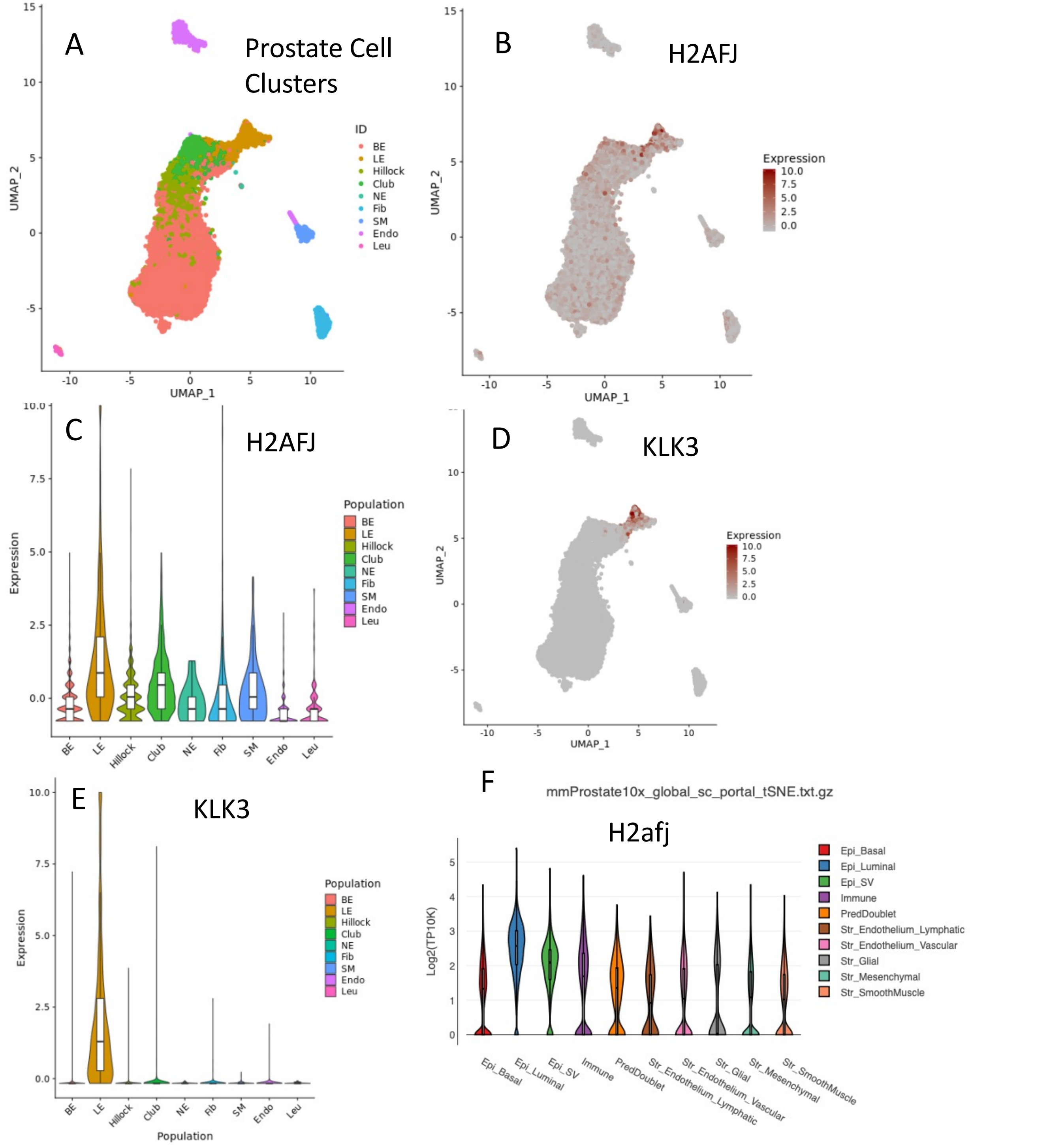

Supplementary Figure S4. Single-cell RNA-seq data for normal human and mouse prostate epithelial cells [24] show preferential expression of H2AFJ in luminal cells as for the KLK3 prostate-specific antigen gene. (A) UMAP of 9 human prostate cell clusters: BE = Basal Epithelial, LE = Luminal Epithelial, Hillock, Club, NE = Neuroendocrine, Fib = Fibroblast, SM = Smooth Muscle, Endo = Endothelial, Leu = Leukocytes. (B) H2AFJ RNA is enriched in the human luminal epithelial (LE) cell cluster. (C) Violin plot of H2AFJ RNA levels indicating highest expression in the human luminal epithelial (LE) cell cluster and next highest expression in Club cells. (D,E) UMAP and violin plot representation showing highly localized expression of KLK3 in the human luminal epithelial (LE) cell cluster. The violon plots (C,E) show the log-normalized expression values, (F) Violin plot of mouse single-cell RNA-seq data showing preferential expression of H2afj RNA in the luminal epithelial cell cluster (Epi_Luminal) as for the human prostate. The other cell clusters are basal epithelial (Epi_Basal), seminal vesicle epithelial (Epi_SV), immune, predicted doublet cells (PredDoublet), stromal lymphatic endothelium (Str_Endothelium_Lymphatic), stromal vascular endothelium (Str_Endothelium_Vascular), stromal glial (Str_Glial), stromal mensenchymal (Str_Mesenchymal), and stromal smooth muscle (Str_SmoothMuscle). The expression scale, Log2(TP10K), is Log2(Transcripts per 10,000).

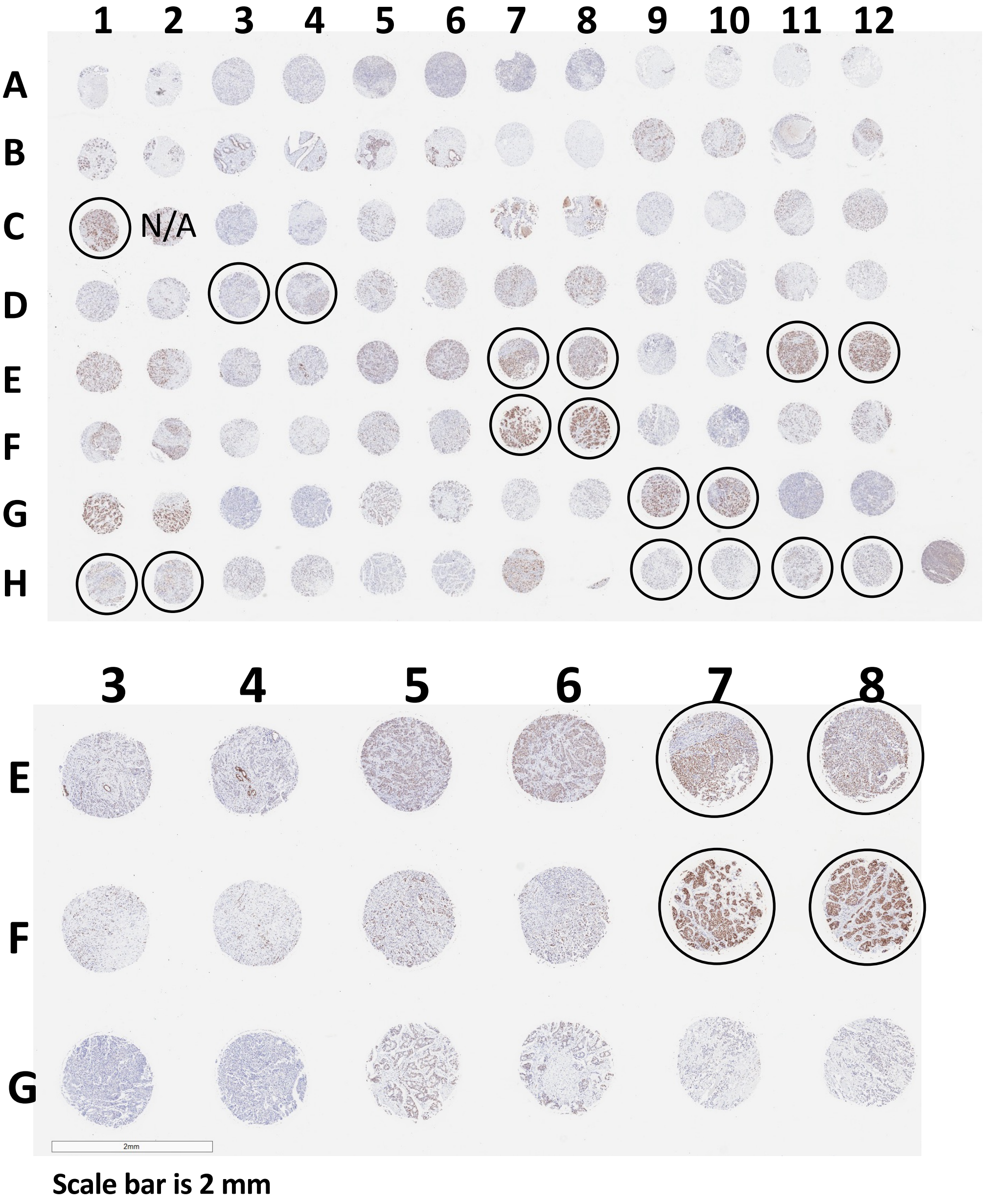

Supplementary Figure S5. H2A.J is expressed at higher average levels in ER-positive breast cancer biopsies compared to ER-negative biopsies. Top, H2FJ staining in a 97–core microarray of normal (A1-A2), inflamed (A3 to A8), benign (A9 to B8) and malignant (B9 to H12) breast tissues. Malignant tissues comprise invasive ductal carcinoma (B9 to H8) and invasive lobular carcinoma (H9 to H12). Circles denote ER positive malignant samples. N/A denotes unknown ER status in one malignant core. All samples are from females aged 27 to 75 years old. Cancer grades in malignant samples are as follow: Grade 1 (B9 to C7, C10 to C12, D5, D6 and E4); Grade 2 (D1 to D4, D7 to E3, E5 to F8, F11 to G2, G5, G6 and H1 to H6) and Grade 3 (F9, F10, G3, G4, G7 to G12, H7 and H8). The H12 core is adrenal gland tissue.
Bottom, enlargement of a central area of the microarray shown above.

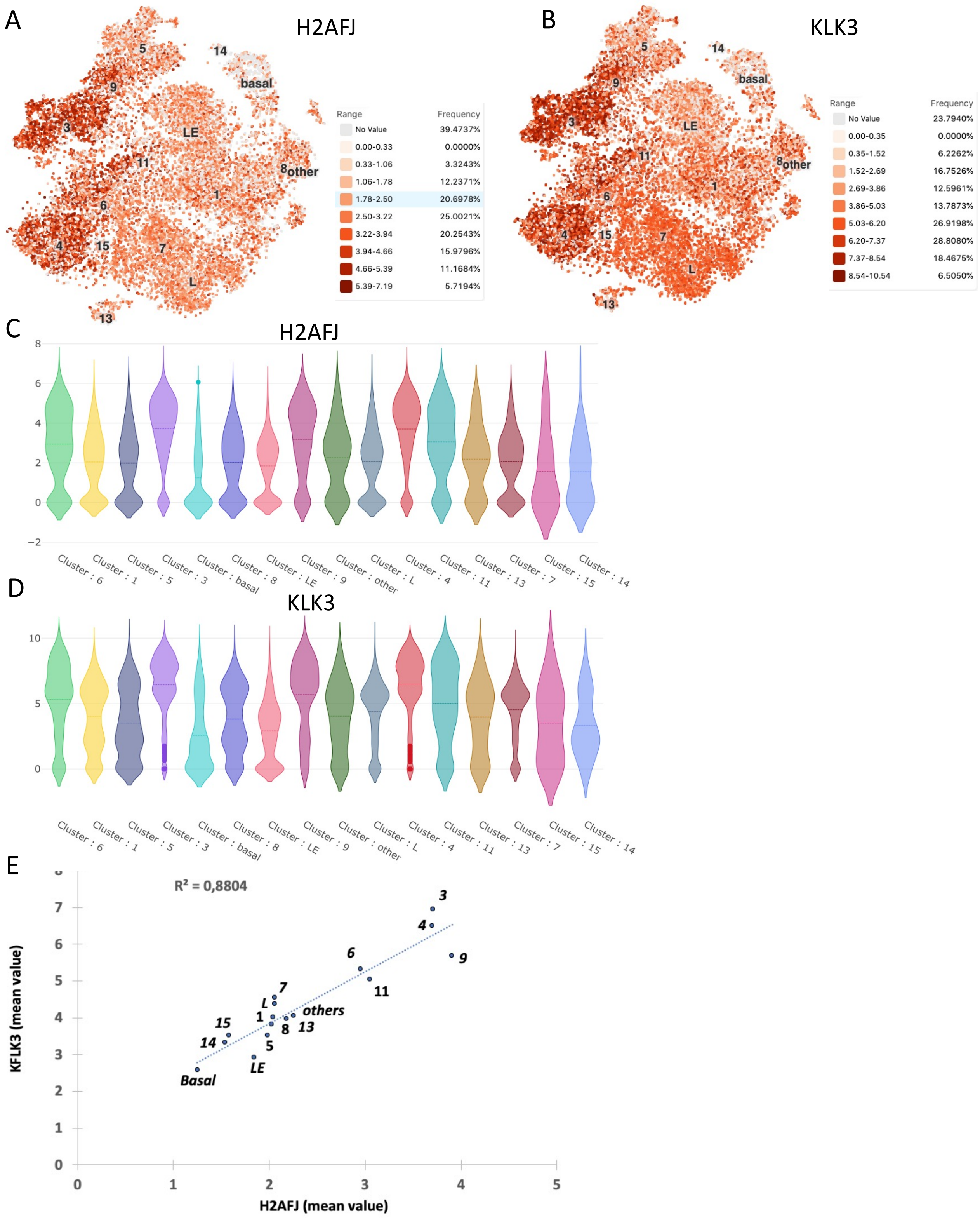

Supplementary Figure S6. Single-cell RNA-seq analysis of prostate cancer epithelial cells shows that H2AFJ (**A,C**) and KLK3 (**B,D**) are expressed in a similar set of luminal-type epithelial prostate cancer cell clusters as shown in UMAP representations and violin plots. (**E**) Plot of mean values of H2AFJ versus KLK3 RNA levels in the prostate cancer clusters shown in (**A,B,C,D**) showing a correlation coefficient (R^2) of 0.88.

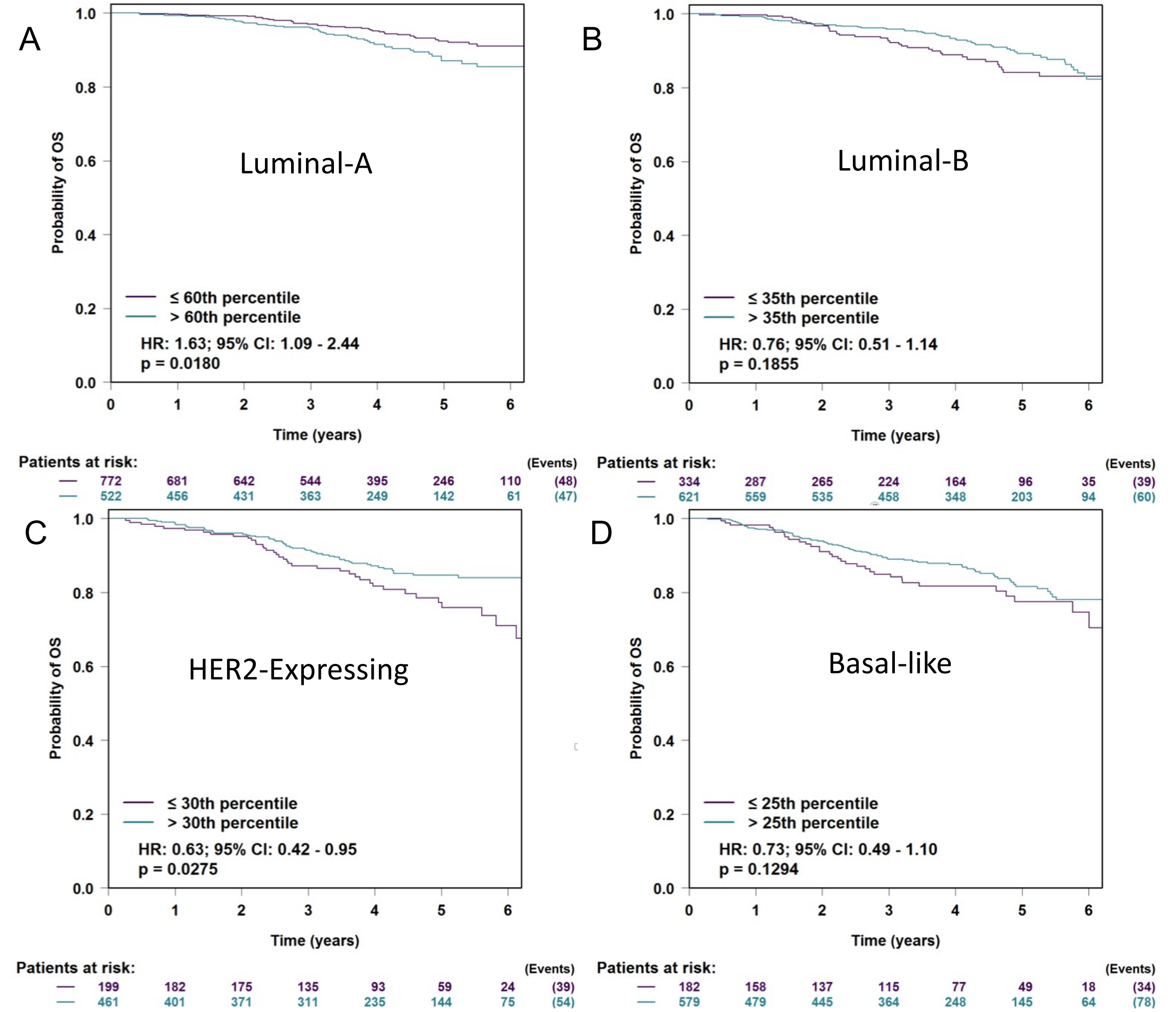

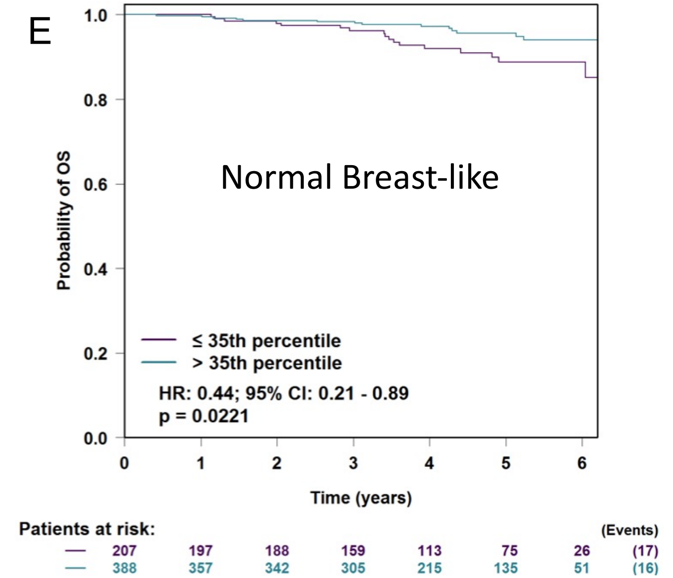

Supplementary Figure S7. Kaplan-Meier survival curves for breast cancer patients expressing the indicated levels of H2AFJ RNAs. High H2AFJ tends to be correlated with better survival for all PAM50 breast cancer subtypes except for Luminal-A for which high H2AFJ expression is correlated with poorer survival.

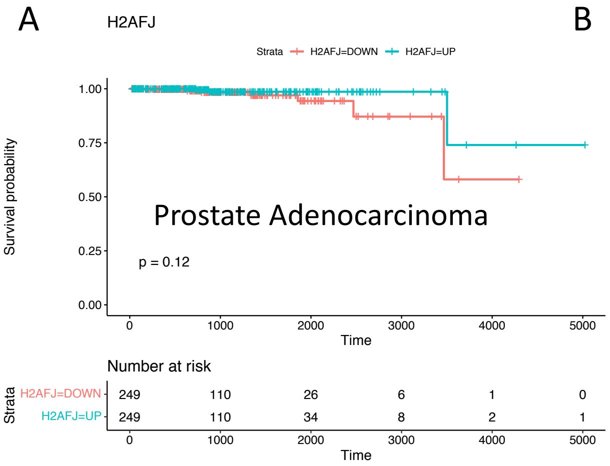

B

Cancer	Cox Coefficient	P-Value	FDR Corrected	Rank	Median Expression
LGG	0.306	6.60e-04	2.46e-03	4513	163.15
KIRC	0.229	2.90e-03	1.19e-02	4045	907.36
SKCM	0.191	4.80e-03	4.00e-02	1924	1284.97
BLCA	-0.215	5.60e-03	8.00e-02	1142	2318.58

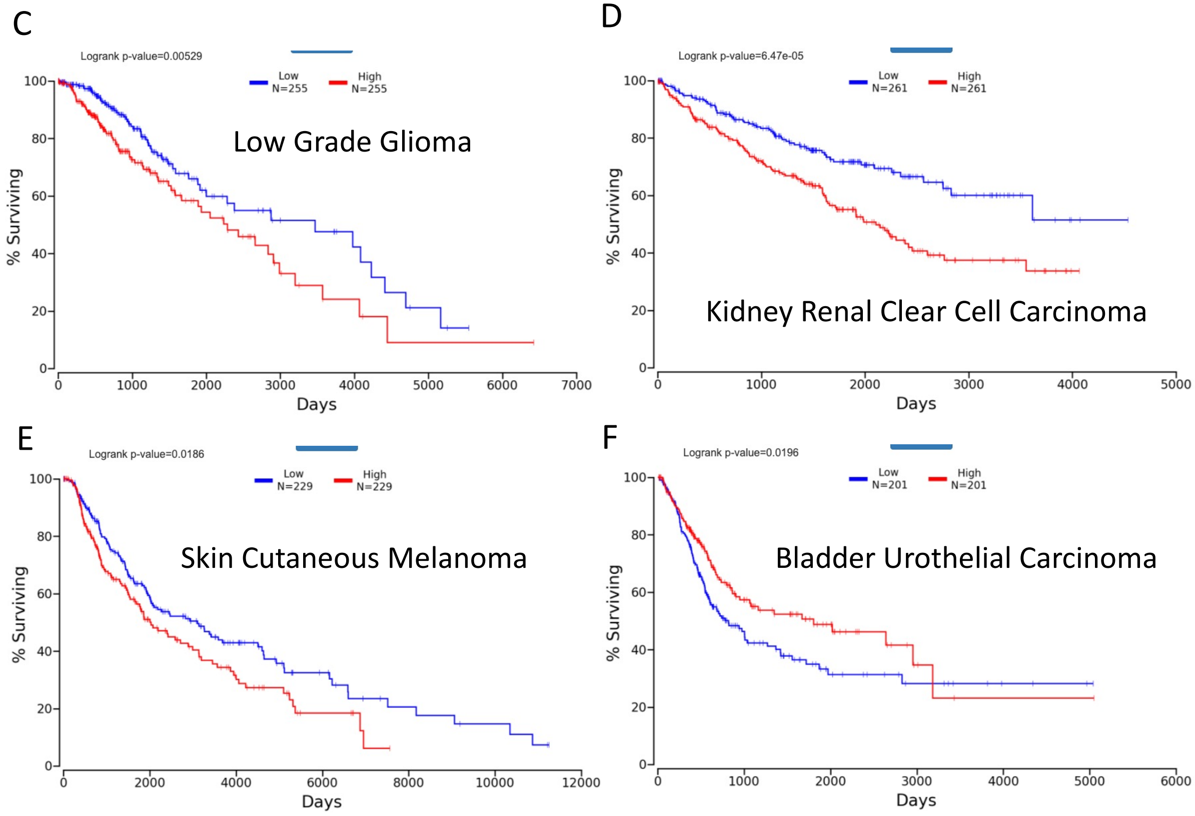

Supplementary Figure S8. Kaplan-Meier survival curves for the indicated TCGA cancer types as a function of H2AFJ RNA expression. In all cases, the comparison involved patients expressing more than the median levels of H2AFJ versus patients expressing less than the median levels of H2AFJ. (**A**) Prostate adenocarcinoma patients expressing more H2AFJ tend to have a better survival than patients expressing less H2AFJ, but this tendency does not reach statistical significance in this cohort in which there were only 10 deaths. (**B**) Table showing the 4 TGCA cancers: (**C**) Low Grade Glioma (LGG), (**D**) kidney renal cell carcinoma (KIRC), (**E**) skin cutaneous melanoma (SKCM), and (**F**) bladder urothelial carcinoma (BLCA), in which H2AFJ expression levels appear to significantly affect the survival of patients as determined by the OncoLnc database [27]. Shown are the Cox correlation coefficient, p-value, FDR-corrected p-value, the rank of the gene in predicting survival, and the median expression (TPM) of H2AFJ RNA in each cancer type. Patients expressing higher H2AFJ have lower survival in LGG, KIRC, and SKCM, but higher survival in BLCA.

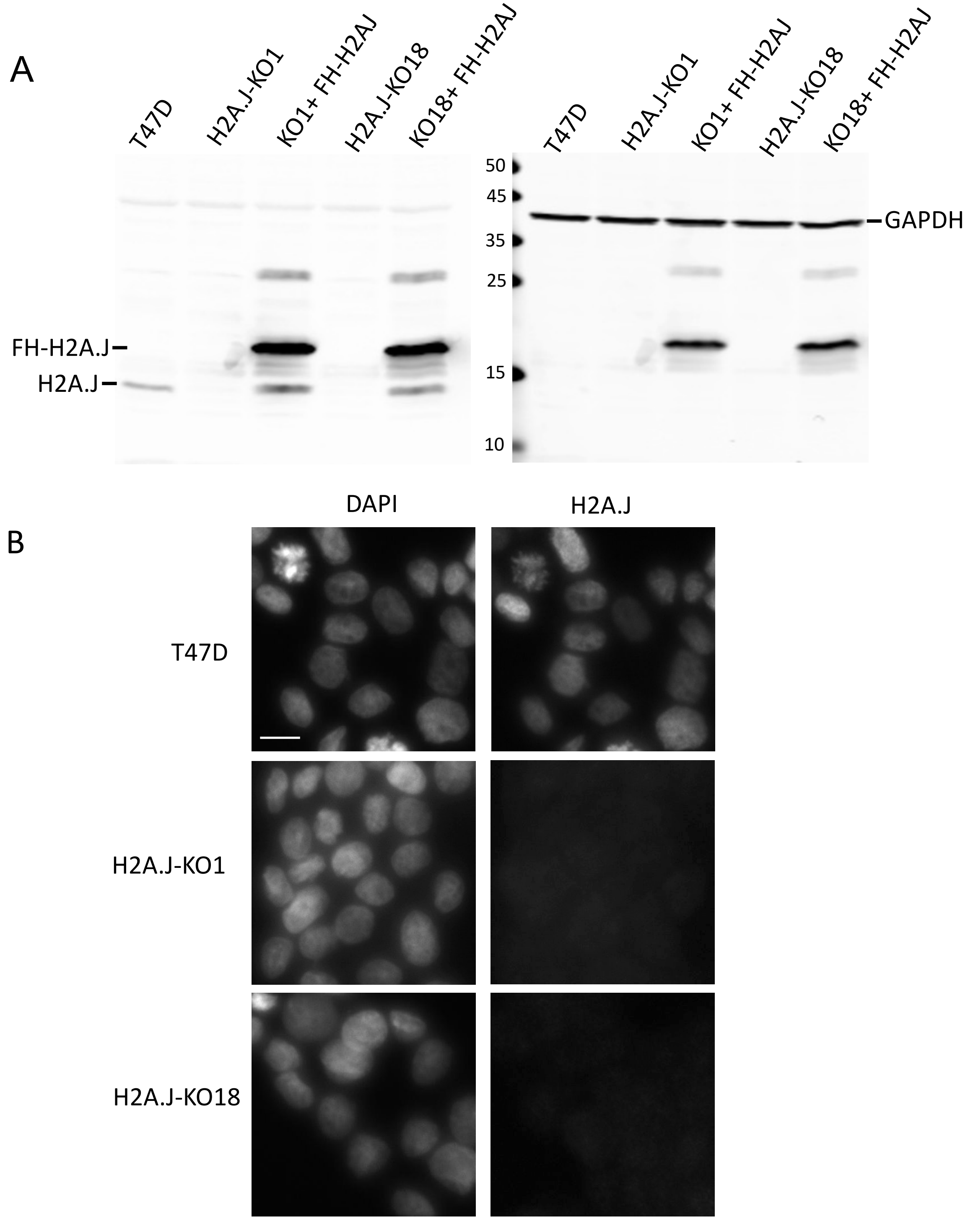

Supplementary Figure S9. Absence of H2A.J expression in T47D-H2A.J-KO1 and T47D-H2A.J-KO18 cell lines compared to parental T47D cells as determined by Western blotting (**A**) and immunofluorescence (**B**) with an anti-H2A.J antibody. Also shown in (**A**) are protein extracts from H2A.J-KO cell lines expressing a Flag-HA-H2A.J (FH-H2A.J) cDNA from a tet-ON promoter. The scale bar for the immunofluorescent images indicates 5 µm.

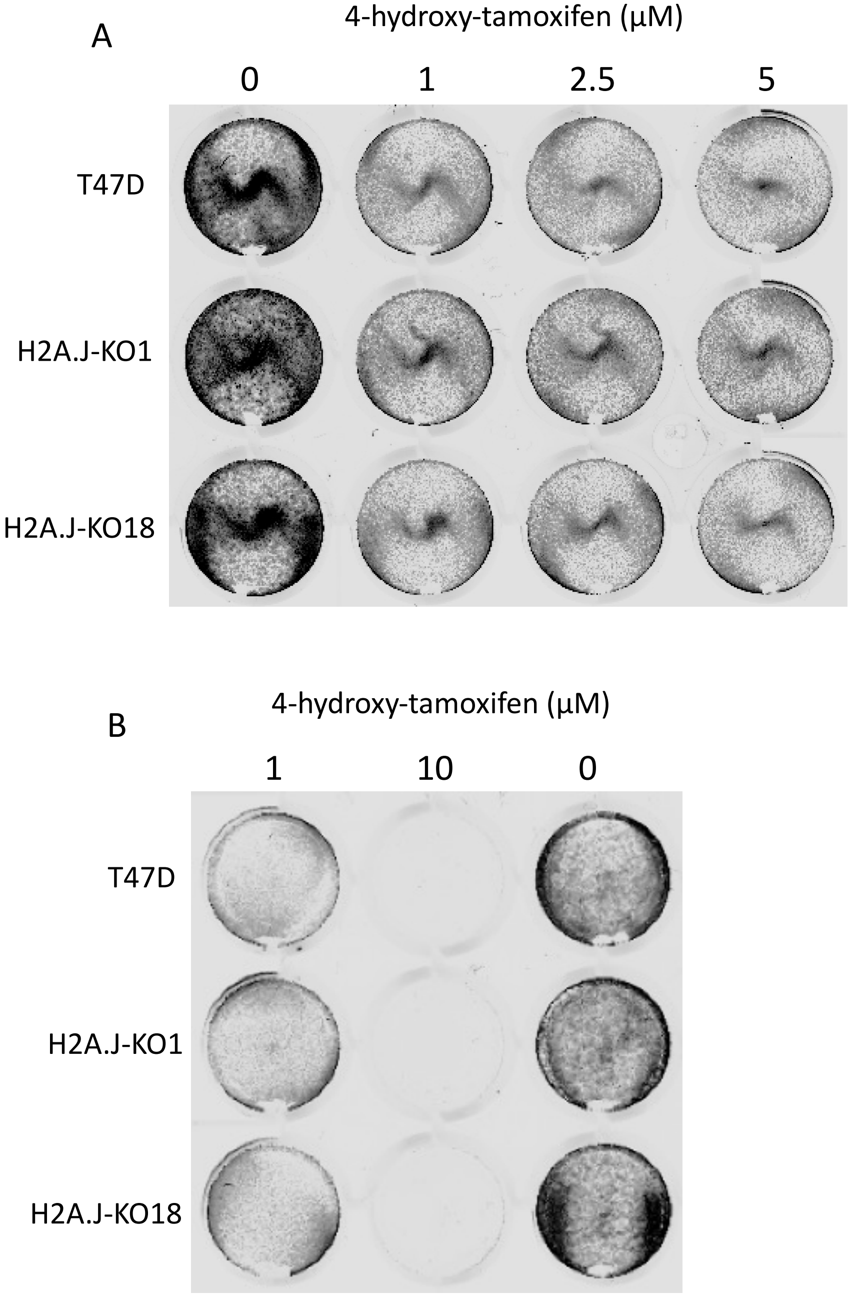

Supplementary Figure S10. T47D, T47D-H2A.J-KO1, and T47D-H2A.J-KO18 cells show similar sensitivity to growth inhibition by 4-hydroxy-tamoxifen. 50,000 cells of each genotype were plated in the wells of a 24-well plate for 2 days before adding 4-hydroxy-tamoxifen at the indicated doses and incubating for an additional 7 days. Cells were then fixed in formaldehyde and stained with Crystal Violet to visualize cell growth on the plates.

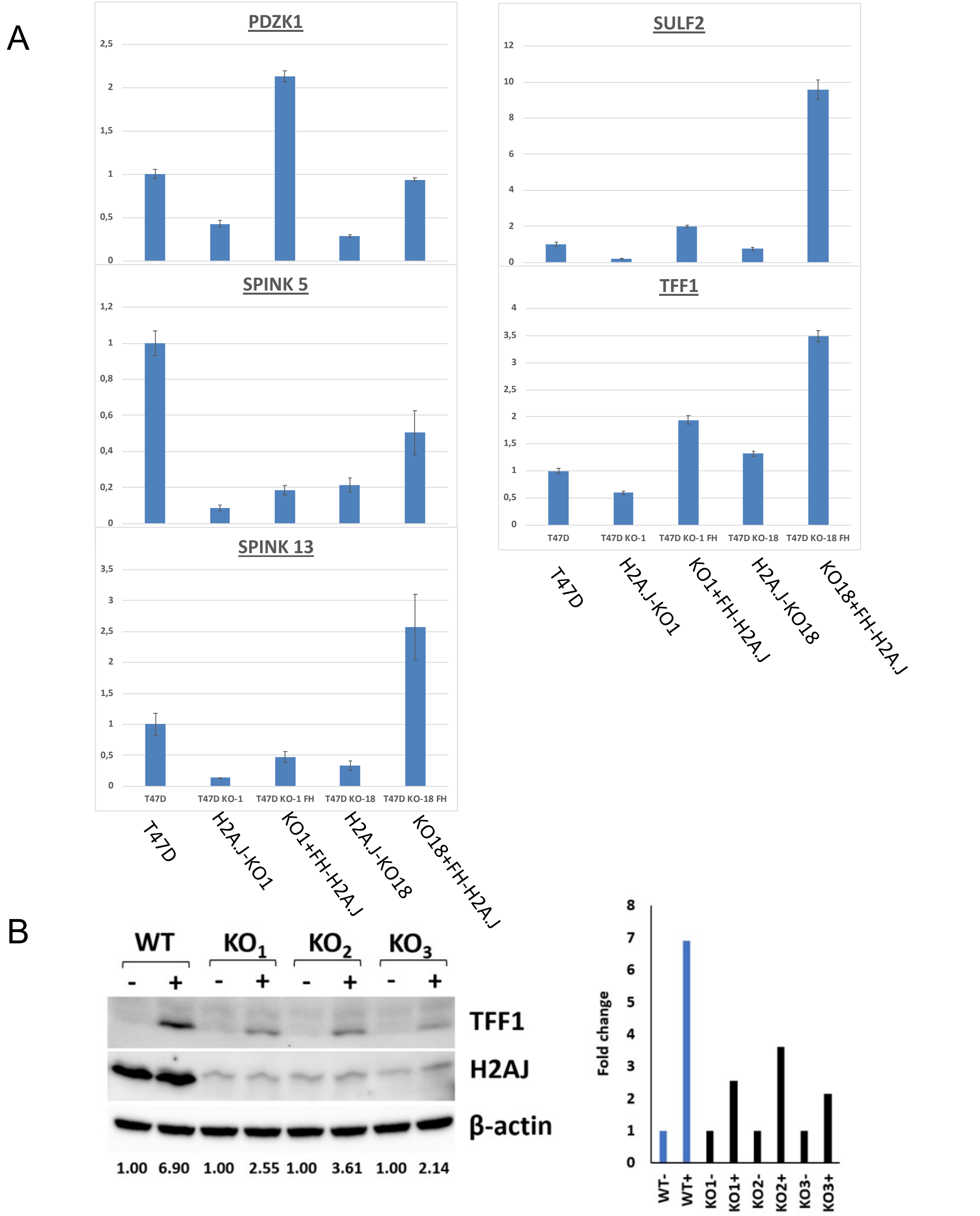

Supplementary Figure S11. Expression of estrogen-responsive genes is inhibited in H2A.J-KO cell lines relative to parental T47D cells. (**A**) RT-qPCR expression analysis of the indicated genes in T47D, H2A.J-KO1, H2A.J-KO18 cells, and in the H2A.J-KO cells after expression of a Flag-HA-H2A.J (FH-H2A.J) cDNA from the tet-ON promoter (Supplementary Figure 8). (**B**) TFF1 Western bots from T47D or 3 independent H2A.J-KO cells lines that were grown in medium depleted for estrogen (-) or after addition of 10 nM estradiol for 48 hours (+). Numbers under the panels represent the intensity ratios for H2A.J/ ß-actin normalized to the untreated T47D ratio.

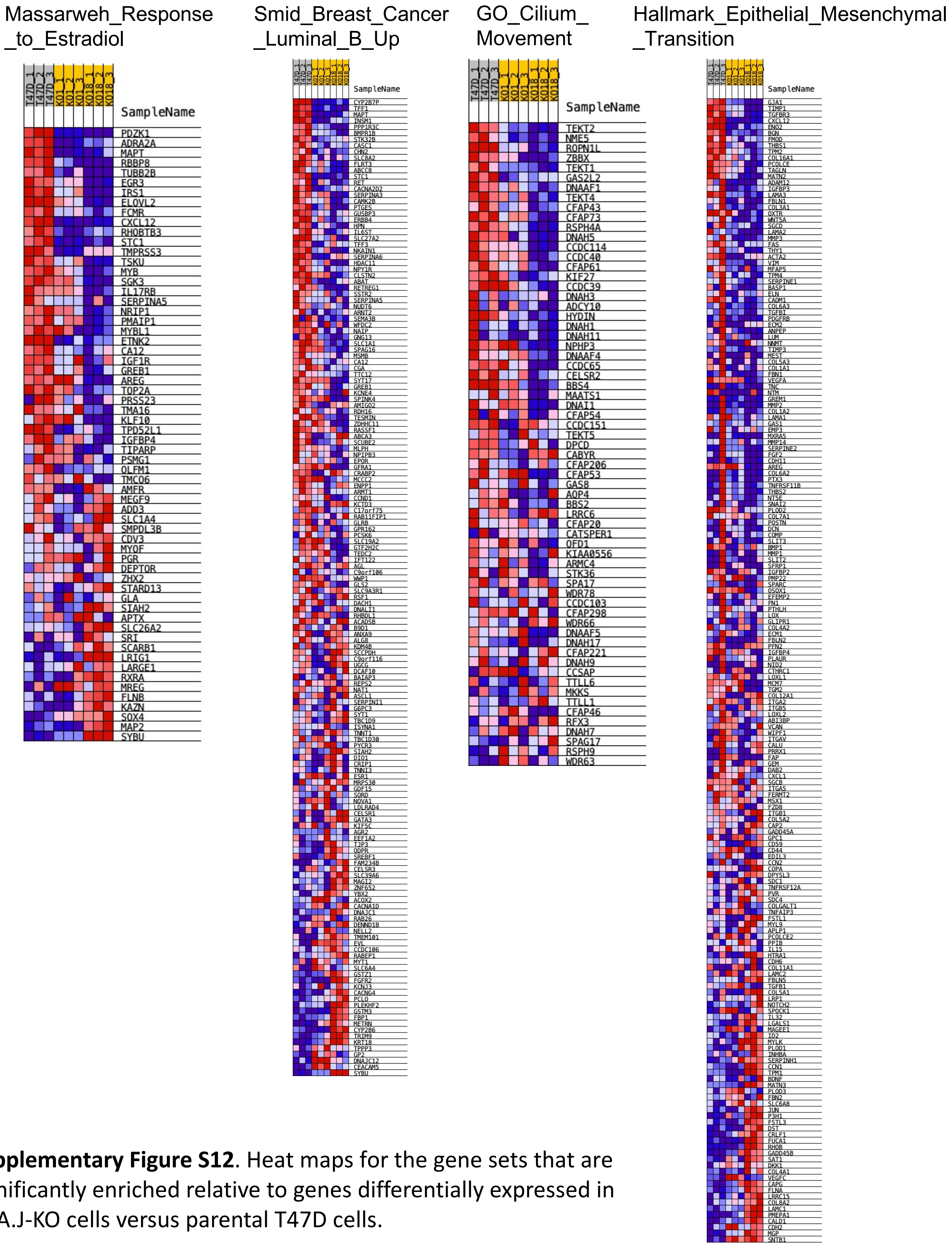

Supplementary Figure S12. Heat maps for the gene sets that are significantly enriched relative to genes differentially expressed in H2A.J-KO cells versus parental T47D cells.
