# Supplementary material for: Histone Variant H2A.J Is Enriched in Luminal Epithelial Gland Cells
Source: Genes (Basel). 2021 Oct 22;12(11):1665. doi: 10.3390/genes12111665 (PMC8619693; doi:10.3390/genes12111665)
Supplement: Supplementary file 1 [file genes-12-01665-s001.zip › SupplementaryTable1.pdf]

Supplementary Table S1. Number of patient samples for each tissue type obtained from the TGEx v.8 database.

Tissue	Number of samples
Adipose Tissue	1204
Adrenal Gland	258
Bladder	21
Blood	929
Brain	2642
Breast	459
Colon	779
Esophagus	1445
Fallopian Tube	9
Heart	861
Kidney	89
Liver	226
Lung	578
Muscle	803
Ovary	180
Pancreas	328
Pituitary	283
Prostate	245
Salivary Gland	162
Skin	1809
Small Intestine	187
Spleen	241
Stomach	359
Testis	361
Thyroid	653
Uterus	142
Vagina	156
